# Comparative genome analysis of novel coronavirus (SARS-CoV-2) from different geographical locations and the effect of mutations on major target proteins: An *in silico* insight

**DOI:** 10.1371/journal.pone.0238344

**Published:** 2020-09-03

**Authors:** Mohd Imran Khan, Zainul A. Khan, Mohammad Hassan Baig, Irfan Ahmad, Abd-ElAziem Farouk, Young Goo Song, Jae-Jun Dong

**Affiliations:** 1 Department of Biophysics, All India Institute of Medical Sciences, New Delhi, India; 2 Department of Plant Molecular Biology, University of Delhi South Campus, New Delhi, India; 3 Department of Family Medicine, Yonsei University College of Medicine, Seoul, Republic of Korea; 4 Department of Clinical Laboratory Sciences, College of Applied Medical Sciences, King Khalid University, Abha, Saudi Arabia; 5 Research Center for Advanced Materials Science, King Khalid University, Abha, Saudi Arabia; 6 Department of Biotechnology, Faculty of Science, Taif University, Taif, Saudi Arabia; 7 Department of Infectious Diseases, Gangnam Severance Hospital, Yonsei University College of Medicine, Seoul, South Korea; King Abdulaziz University, SAUDI ARABIA

## Abstract

A novel severe acute respiratory syndrome-related coronavirus-2 (SARS-CoV-2) causing COVID-19 pandemic in humans, recently emerged and has exported in more than 200 countries as a result of rapid spread. In this study, we have made an attempt to investigate the SARS-CoV-2 genome reported from 13 different countries, identification of mutations in major coronavirus proteins of these different SARS-CoV-2 genomes and compared with SARS-CoV. These thirteen complete genome sequences of SARS-CoV-2 showed high identity (>99%) to each other, while they shared 82% identity with SARS-CoV. Here, we performed a very systematic mutational analysis of SARS-CoV-2 genomes from different geographical locations, which enabled us to identify numerous unique features of this viral genome. This includes several important country-specific unique mutations in the major proteins of SARS-CoV-2 namely, replicase polyprotein, spike glycoprotein, envelope protein and nucleocapsid protein. Indian strain showed mutation in spike glycoprotein at R408I and in replicase polyprotein at I671T, P2144S and A2798V,. While the spike protein of Spain & South Korea carried F797C and S221W mutation, respectively. Likewise, several important country specific mutations were analyzed. The effect of mutations of these major proteins were also investigated using various *in silico* approaches. Main protease (Mpro), the therapeutic target protein of SARS with maximum reported inhibitors, was thoroughly investigated and the effect of mutation on the binding affinity and structural dynamics of Mpro was studied. It was found that the R60C mutation in Mpro affects the protein dynamics, thereby, affecting the binding of inhibitor within its active site. The implications of mutation on structural characteristics were determined. The information provided in this manuscript holds great potential in further scientific research towards the design of potential vaccine candidates/small molecular inhibitor against COVID19.

## Introduction

In the last two decades, three coronaviruses *viz*. severe acute respiratory syndrome coronavirus (SARS-CoV) [1], Middle-East respiratory syndrome coronavirus (MERS-CoV) [2] and SARS-CoV-2 have crossed the species barrier to cause deadly pneumonia in humans. In 2002, SARS-CoV emerged in the Guangdong province of China and spread to five continents, infecting 8,098 people with 774 deaths. In 2012, MERS-CoV emerged in the Arabian Peninsula, transmitted to 27 countries, infecting a total of ~2,494 individuals and claiming 858 lives. The current outbreak of coronavirus disease 19 (COVID-19) caused by SARS-CoV-2, was first reported in December 2019 in Wuhan, Hubei province of China [3, 4] and spread across 200 countries, infecting over 2.5 million people and killed more than 1.5 lakh as of April, 23, 2020. SARS-CoV-2 was declared a pandemic by the World Health Organization on March 12, 2020.

**Fig 1:**
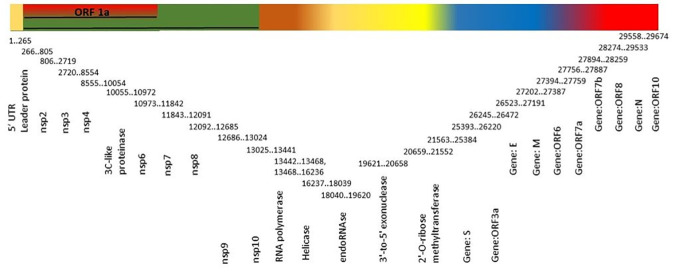
Schematic representation of genome organization of SARS-CoV-2.

SARS-CoV-2 belongs to the family *Coronaviridae* of genus *Betacoronavirus*, having positive sense strand RNA genome of 26–32 kb size. SARS-CoV-2 genome has six major open reading frames (ORFs) *viz*. replication enzyme coding region (ORF 1a and 1b), E gene (envelope protein), M gene (membrane protein), S gene (spike protein), and N gene (nucleocapsid protein) that are common to coronaviruses and a number of other accessory genes (ORF 3a, 6, 7a, 7b and 8) (Fig 1) [3]. The structural proteins: envelope protein, nucleocapsid protein, spike protein and membrane protein are essential for producing the structurally complete viral particle [5–8]. Entry of coronavirus into host cells is guided by spike glycoprotein. ORF 1a and 1b encode replication enzyme consisting 16 non-structural proteins (nsp1-16) that are highly conserved among the coronaviruses. Main protease (Mpro, also known as 3CLpro) is one of the important nsp encoded by ORF 1a and 1b, play an essential role in the processing of polyproteins and control the replication of coronavirus [9, 10]. RNA-dependent RNA polymerase (RdRp) also known as nsp12, another important replicase catalyze the replication of RNA using viral genomic RNA template [11].

Reports showed that MERS-CoV originated from bats, but the reservoir host fueling spillover to humans is unequivocally dromedary camels [12, 13]. Both SARS-CoV and SARS-CoV-2 are originated from bats, which serve as reservoir host for these two viruses [3, 14, 15]. Raccoon dogs and palm civets have been identified as intermediate hosts for zoonotic transmission of SARS-CoV between bats and humans [16–18], however, the intermediate host of SARS-CoV-2 remains unknown.

Mutation rate is very high in RNA viruses, up to a million times higher than their host, which enhance their virulence and evolvability (formation of new species) [19]. Coronavirus replication is error prone as compared to other RNA viruses and the estimated mutation rate is 4x10^-4^ nucleotide substitutions/site/year [20]. The rate of SARS-CoV-2 mediated disease spread and the mortality varies from country to country. Of several reasons affecting the rate of disease spread and mortality, mutations within the SARS-CoV-2 strains is also considered one of the major factors.

This study was conducted to gather additional information on the SARS-CoV-2 sequences from different geographical locations infected with COVID-19. The genome analysis of the SARS-CoV-2 strains from 13 different countries showed a large number of mutations within the major structural proteins. This is the first time we have comprehensively investigated these mutations and also discussed their potential roles in the pathogenicity, replication and entry of virus particle. This study provides a deeper insight into the emergence of these mutations within the major structural as well as nsp encoded by the SARS-CoV-2 genome from different countries. Here, molecular dynamics and other *in silico* studies were also performed to investigate the effect of mutations on the dynamics of Mpro. The findings of this study provide a clue for the futuristic development of potential vaccine candidate or therapeutic design against COVID19.

## Material and methods

### Sequence analysis and stability prediction

The genome sequence of ORF1ab for SARS with reference sequence ID: NC_004718.3 and protein sequence with GenBank ID: AAP41036.1, was retrieved from NCBI database. Similarly, the genome sequence for SARS-CoV-2 with Reference Sequence ID: MT012098.1, MT019529.1, MT039890.1, MT093571.1, MT192772.1, MT126808.1, MT192759.1, MN985325.1, MT007544.1, LC529905.1, MT020781.2, MT072688.1 and MT066156.1 and protein sequence with GenBank ID: QHS34545.1, QHU36823.1, QHZ00378.1, QIC53203.1, QIK50437.1, QIG55993.1, QIK50416.1, QHO60603.1, QHR84448.1, BCB15089.1, QHU79171.2, QIB84672.1 QIA98553.1 for India, China, South-Korea, Sweden, Vietnam, Brazil, Taiwan, USA, Australia, Japan, Finland, Nepal and Italy, respectively, were downloaded from NCBI. Multiple sequence alignment (MSA) and visualization of SARS and SARS-CoV-2 sequences from 13 different countries was performed using Molecular Evolutionary Genetics Analysis (MEGA) version 10.1.8. To delineate and analyze the mutation across different countries, an in-house script written in Perl and Python was used. MUPRO server was used to determine the effect of mutation on various SARS-CoV-2 proteins [21].

### Model building

The crystal structure of Mpro protein from SARS-CoV-2 in complex with Boceprevir (pdb id: 7BRP) was taken as a wildtype (WT). The structure of R60C was generated by inserting the Point mutation and modelled using modeler 9v13 [22].

### Molecular docking

Boceprevir was retrieved and redocked within the structure of Mpro using CCDC Gold. The RMSD for the crystal and redocked conformation of Boceprevir were compared. Further, Boceprevir was subjected to dock within the active site of R60C mutant. The poses were visualized on PMV viewer [23].

### Molecular dynamics simulations of the protein and their complexes

The structure of Boceprevir in complex with Mpro (WT) and R60C mutant was subjected to energy minimization using Gromacs-5 with the CHARMM27 all atom force field [24–26]. The models were solvated with a SPC/E water model in a cubic periodic box with 1 nm distance from the edge of the complex atoms. The solvated system was neutralized by seven chloride ions. The system was, thereafter, minimized using steepest descent algorithm with convergence criteria of tolerance value 1000 kJ mol^−1^ nm^−2^. The complete simulation of minimized solvated proteins was performed under periodic boundary condition with time step of 2 fs. Particle mesh Ewald was used for long range electrostatic interactions with an interpolation order of 4 and a Fourier spacing of 0.16. The first phase simulation was conducted under an NVT ensemble for 500 ps by keeping all bonds constrained using the LINCS algorithm for temperature equilibration. The system was heated to 300 K using leap-frog integrator while pressure coupling was set off. A V-rescale thermostat was used to maintain constant temperature for each system, followed by pressure equilibration at 300 K using Parrinello-Rahman pressure coupling algorithm under an isothermal-isobaric ensemble for another 500 ps at 1.0 bar. Isothermal compressibility of the solvent was set to 4.5e^-5^ bar^−1^. Further, simulation of 50000 ps production run was carried out at 300 K and 1 atm pressure for trajectory analysis. The final models obtained at the end of MD were validated and taken for structural analysis.

## Results

### Sequence analysis and mutation detection

**Fig 2:**
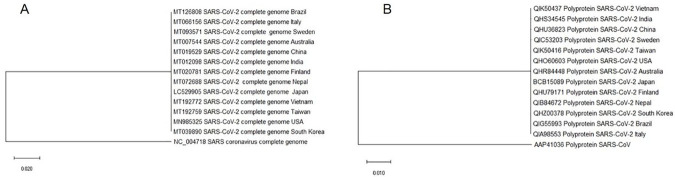
Phylogenetic dendogram showing the relationship of SARS-CoV-2 complete sequence (nucleotide) from different geographical locations (13 no.) with SARS-CoV (A) and amino acid sequence of replicase polyprotein of 13 SARS-CoV-2 with SARS-CoV (B). The evolutionary history was inferred using the Neighbor-Joining method.

The complete nucleotide sequences of 13 SARS-CoV-2 reported from 13 different countries showed ~82% sequence identity with SARS-CoV. Also, all 13 sequences shared more than 99% sequence identity to each other. Replicase polyprotein (ORF 1ab) of 13 isolates, which are most conserved in all coronaviruses shared maximum identity (87%) with SARS-CoV (NC_0047180), which is less than the threshold value (90%) for demarcation of betacoronavirus species [27, 28]. Phylogenetic analysis revealed that all 13 SARS-CoV-2 identified from different geographical locations clustered together in a single clad as compared to SARS-CoV (Fig 2A and 2B).

Further, we checked the mutation in all major proteins of 13 SARS-CoV-2 sequences and compared with SARS-CoV. ORF 1a and 1b showed 11 changes among all 13 SARS-CoV-2. Indian SARS-CoV-2 sequence showed three changes at 671 (Isoleucine to Threonine), 2144 (Proline to Serine) and 2798 (Alanine to Valine) compared to all other 12 isolates. Here, we also noted two amino acid mutations (in ORF1ab) in each SARS-CoV-2 sequences isolated from China (2708: Asparagine to Serine; 2908: Phenylalanine to Isoleucine), South Korea (902: Methionine to Isoleucine; 6891: Threonine to Methionine) and Sweden (818: Glycine to Serine; 4321: Phenylalanine to Leucine). Brazil and Vietnam isolate showed only one change at 3606 (Leucine to Phenylalanine) and 3323 (Arginine to Cystine), respectively (Table 1). 996 changes have been reported when ORF 1a and 1b amino acid sequences of all 13 SARS-CoV-2 was compared with SARS-CoV (S1 File).

**Table 1:** Amino acid variation in replicase polyprotein of SARS-CoV-2 strains of 13 different countries.

Amino acid	India	China	South Korea	Sweden	Vietnam	Brazil	Taiwan	USA	Australia	Japan	Finland	Nepal	Italy
671	T	I	I	I	I	I	I	I	I	I	I	I	I
818	G	G	G	S	G	G	G	G	G	G	G	G	G
902	M	M	I	M	M	M	M	M	M	M	M	M	M
2144	S	P	P	P	P	P	P	P	P	P	P	P	P
2708	N	S	N	N	N	N	N	N	N	N	N	N	N
2908	F	I	F	F	F	F	F	F	F	F	F	F	F
3323	R	R	R	R	C	R	R	R	R	R	R	R	R
3606	L	L	L	L	L	F	L	L	L	L	L	L	X
4321	F	F	F	L	F	F	F	F	F	F	F	F	F
4798	V	A	A	A	A	A	A	A	A	A	A	A	A
6891	T	T	M	T	T	T	T	T	T	T	T	T	T

**Fig 3:**
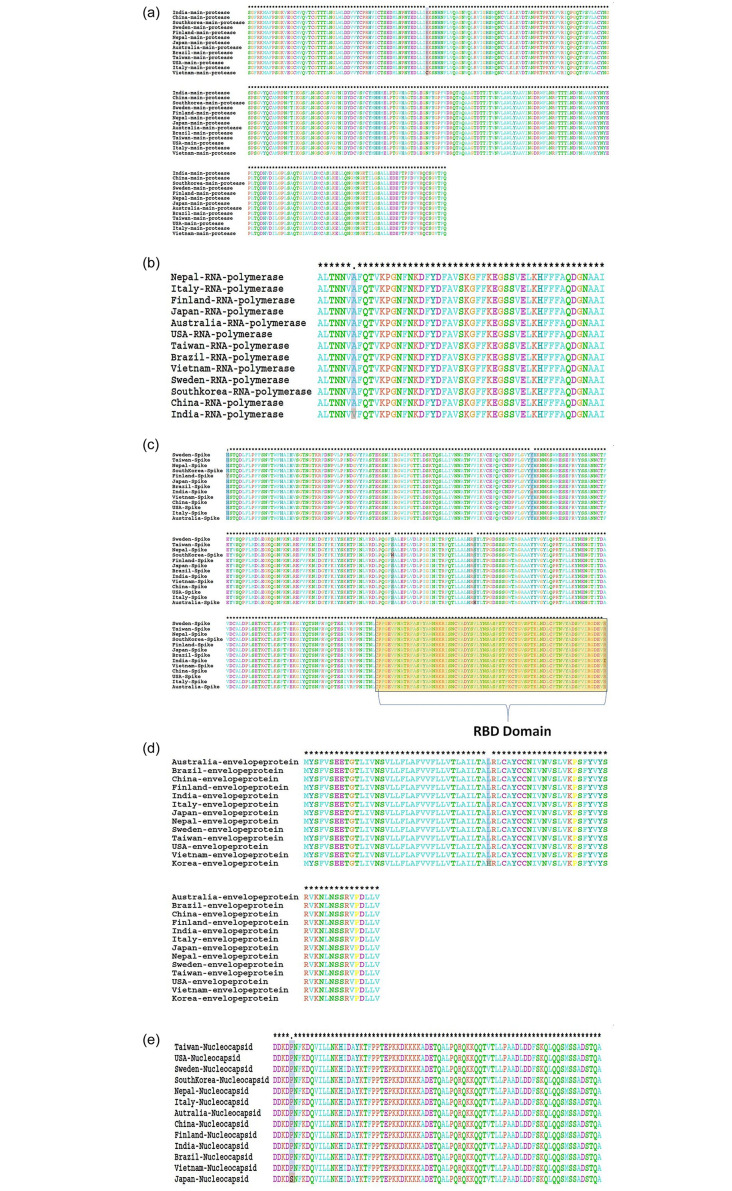
Alignment of SARS-CoV-2 major proteins (A) main protease, (B) RNA-dependent RNA polymerase, (C) spike proteins, (D) envelope proteins and (E) nucleocapsid proteins from different countries.

The viral Mpro controls the replication of coronavirus and is a key protein responsible for its life cycle [29–31]. Mpro is an attractive drug discovery target. The analysis of Mpro reveals that there was only one point mutation (R60C) in the Vietnam strain of SARS-CoV-2 (Fig 3A). RdRp, which is another important target for antiviral drugs functions by catalyzing the viral RNA synthesis [32]. Only one mutation (A406V) was observed in the RdRp of Indian SARS-CoV-2 isolate (Fig 3B).

Spike proteins are the key surface glycoproteins and are well reported for their prominent role in interaction with host cell receptors [33, 34]. Here, we analyzed the mutations in the spike protein of SARS-CoV-2 from different countries. It was found that this glycoprotein carried five different amino acid mutations at various positions within the investigated SARS-CoV-2 isolates. For instance, India, Finland, Australia, South Korea and Sweden SARS-CoV-2 isolates showed one amino acid change at 408 (Arginine to Isoleucine), 49 (Histidine to Tyrosine), 247 (Serine to Arginine), 221 (Serine to Tryptophan) and 797 (Phenylalanine to Cysteine), respectively (Table 2 and Fig 3C). The value of ΔΔG show that the mutant R408I (0.49732107 kcal/mol) mutation was having stabilization effect on spike protein. It was found that the mutation on the receptor binding domain (RBD) of spike protein increases the stability.

**Table 2:** Amino acid variation in spike protein of SARS-CoV-2 strains of 13 different countries.

Amino acid	QHS34546 India	QHU79173 Finland	QHR84449 Australia	QHZ00379 South Korea	QIG55994 Brazil	QIK50438 Vietnam	QIK50417 Taiwan	QIA98554 Italy	QHU36824 China	QHO60594 USA	BCB15090 Japan	QIC53204 Sweden	QIB84673 Nepal
49	H	Y	H	H	H	H	H	H	H	H	H	H	H
145	-	Y	Y	Y	Y	Y	Y	Y	Y	Y	Y	Y	Y
221	S	S	S	W	S	S	S	S	S	S	S	S	S
247	S	S	R	S	S	S	S	S	S	S	S	S	S
408	I	R	R	R	R	R	R	R	R	R	R	R	R
797	F	F	F	F	F	F	F	F	F	F	F	C	F

When these 13 SARS-CoV-2 isolates were compared to SARS-CoV sequence, 1338 changes have been reported (S1 File). The analysis of ORF3a showed 3 mutations within different SARS-CoV-2 strains: W128L (South Korea), L140V (Japan), G251V (Australia, South Korea, Brazil, Italy, Sweden) (Table 3).

**Table 3:** Amino acid variation in ORF 3 encoded protein of SARS-CoV-2 strains of 13 different countries.

Position Amino acid	QHS34546 India	QHU79173 Finland	QHR84449 Australia	QHZ00379 South Korea	QIG55994 Brazil	QIK50438 Vietnam	QIK50417 Taiwan	QIA98554 Italy	QHU36824 China	QHO60594 USA	BCB15090 Japan	QIC53204 Sweden	QIB84673 Nepal
128	W	W	W	L	W	W	W	W	W	W	W	W	W
140	L	L	L	L	L	L	L	L	L	L	V	L	L
251	G	G	V	V	V	G	G	V	G	G	G	V	G

One amino acid change occurred in each envelope protein of South Korea SARS-CoV-2 isolate at 37 (Leucine to Histidine) and nucleocapsid protein of Japan SARS-CoV-2 isolate at 344 (Proline to Serine) when compared among 13 SARS-CoV-2 isolates (Tables 4 and 5, Fig 3D and 3E), while, 5 and 45 changes has been reported in envelop and nucleocapsid proteins, respectively as compared to SARS-CoV. Deletion of Glycine and Serine occurred at position 70 and 8 in envelop and nucleocapsid proteins, respectively, in all 13 SARS-CoV-2 isolates when compared to SARS-CoV. MEM glycoprotein did not show any amino acid change among 13 SARS-CoV-2 isolates, while 24 changes occurred when compared to SARS-CoV (S1 File). All the other point mutations occurring within the structural proteins of SARS-CoV-2 isolates from different countries were found to decrease protein stability (Table 6).

**Table 4:** Amino acid variation in envelop protein of SARS-CoV-2 strains of 13 different countries.

Position Amino acid	QHS34546 India	QHU79173 Finland	QHR84449 Australia	QHZ00379 South Korea	QIG55994 Brazil	QIK50438 Vietnam	QIK50417 Taiwan	QIA98554 Italy	QHU36824 China	QHO60594 USA	BCB15090 Japan	QIC53204 Sweden	QIB84673 Nepal
37	L	L	L	H	L	L	L	L	L	L	L	L	L

**Table 5:** Amino acid variation in nucleocapsid protein of SARS-CoV-2 strains of 13 different countries.

Position Amino acid	QHS34546 India	QHU79173 Finland	QHR84449 Australia	QHZ00379 South Korea	QIG55994 Brazil	QIK50438 Vietnam	QIK50417 Taiwan	QIA98554 Italy	QHU36824 China	QHO60594 USA	BCB15090 Japan	QIC53204 Sweden	QIB84673 Nepal
344	P	P	P	P	P	P	P	P	P	P	S	P	P

**Table 6:** Mutation in SARS-CoV-2 proteins from different geographical locations and their predicted effect on protein stability.

Protein Name	Mutation	Country	Stability effect (MUPRO)
3C-like proteinase (3CLpro)	R60C	Vietnam	DECREASE stability (ΔΔG -1.0163868)
Envelope Protein	L37H	South Korea	DECREASE stability (ΔΔG -2.4215632)
ORF3a	W128L	South Korea	DECREASE stability (ΔΔG -0.39593766)
	L140V	Japan	DECREASE stability (ΔΔG -0.90740107)
	G251V	Australia, South Korea, Brazil, Italy, Sweden	DECREASE stability (ΔΔG -0.45128408)
Spike Protein	H49Y	Finland	DECREASE stability (ΔΔG -0.20900128)
	S221W	Brazil	DECREASE stability (ΔΔG -0.45085799)
	S247R	Australia	DECREASE stability (ΔΔG -1.3464875)
	R408I	India	INCREASE stability (ΔΔG 0.49732107)
	F797C	Sweden	DECREASE stability (ΔΔG -1.501262)
Nucleocapsid	P344S	Japan	DECREASE stability (ΔΔG -1.2252261)
RNA-dependent RNA polymerase	A406V	India	DECREASE stability (ΔΔG -0.76907034)

### Molecular Dynamics (MD) studies

In the present study, we performed the MD simulations for the Boceprevir bound complexes of SARS-CoV-2 Mpro and its R60C mutant to study the effect of mutation on the protein dynamics.

### Root-Mean-Square Deviation (RMSD)

**Fig 4:**
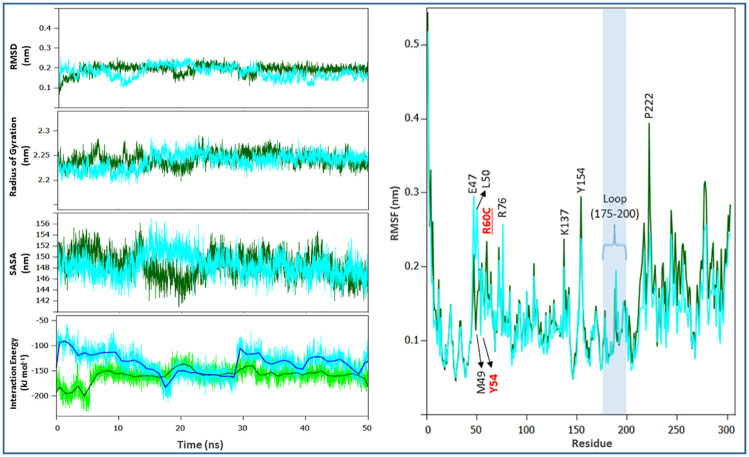
Molecular dynamics of complexed SARS-CoV-2 Mpro and the R60C mutant. Green color indicates the SARS-CoV-2 Mpro while the cyan color indicates the R60C mutant Mpro. (A) Backbone RMSDs of Mpro and its mutated form (B) Rg of Cα atoms (C) Change in Solvent accessible surface area (D) RMSF of the backbone atoms (E) The Lennard–Jones short-range (LJ-SR) and Coulombic short-range (Coul-SR) potential energies.

The root mean square deviations of the backbone were calculated to analyze the trajectories of Mpro from SARS-CoV-2 and its R60C mutant. In the complex form, a slight fluctuation in the backbone RMSD was also noticed (Fig 4A). It was found that the RMSD of mutant was comparatively more stable than its WT. This variation in the average RMSD values suggests that this mutation was affecting the dynamic behavior of Mpro.

### Radius of gyration and SASA

Fig 4B illustrates the Rg of Cα atoms plot of the complexed Mpro from SARS-CoV-2 and the Vietnam mutant Mpro (R60C). The R60C mutant shows slightly lower value for Rg as compared to its WT. This suggests that R60C mutation affects the stability of Mpro. Fig 4C shows the change of SASA of native and R60C mutant with time. The greater value of SASA for R60C mutant (in complexed form) was supported by Rg plot [35].

### Root Mean Square Fluctuation (RMSF)

RMSF values of native as well as R60C mutant Mpro were calculated to determine the impact of mutation on dynamic behavior of protein at residue level. RMSF plot clearly indicates the fluctuation in residues and showed the existence of higher degree of flexibility in R60C mutant Mpro. It was found that the maximum amino acid fluctuation was in the region 50–76 and 127–222 (Fig 4D). The binding studies also confirm that the residues falling within this region were very much involved in accommodating the inhibitor within the active site of Mpro [29, 31].

### Interaction energy and effect on hydrogen bond network

**Fig 5:**
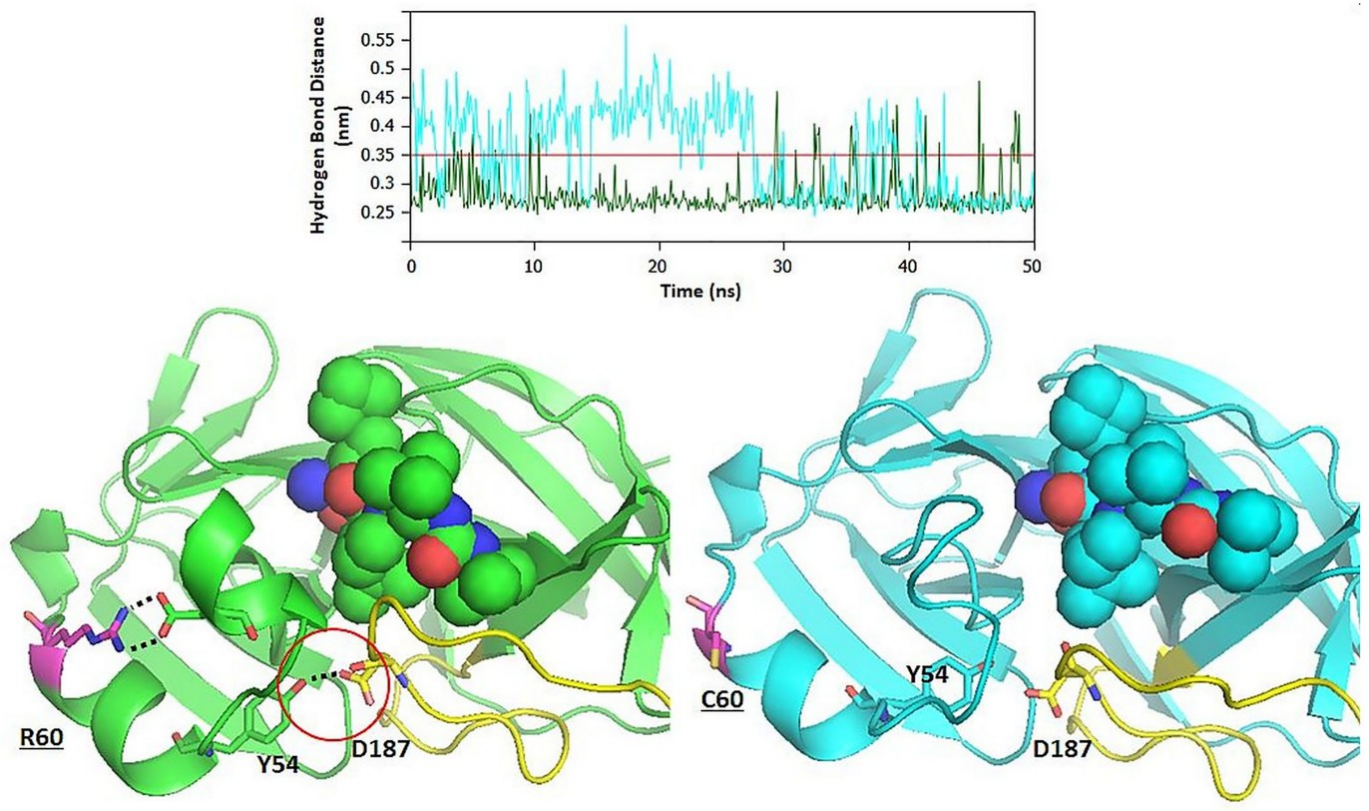
(A) The hydrogen bond network of the Mpro (WT) and R60C mutant. (B) The Structure of WT and R60C mutant Mpro.

Throughout the MD trajectory, the interaction energy of ligand in complex with the surrounding protein residues of WT and mutant Mpro were calculated. The Lennard–Jones short-range (LJ-SR) and Coulombic short-range (Coul-SR) potential energies were calculated throughout the course of 50 ns of MD simulation (Fig 4E). The average interaction energy for 50 ns are shown in Table 7. It was found that the binding of inhibitor within the active site of Mpro (WT) was stronger as compared to the R60C mutant. The analysis of hydrogen bond network revealed that the R60C mutation also cause disturbance in the interactions with inhibitor as well as other surrounding active site residues of Mpro. A large fluctuation was noticed in the hydrogen bond network of Mpro and its R60C mutant (Fig 5A). It was found that R60C mutation results in the changes in local environment that cascade further to the short helix and loop of the catalytic active site of Mpro.

**Table 7:** The Lennard–Jones short-range (LJ-SR) and Coulombic short-range (Coul-SR) potential energies calculated throughout the course of 50 ns of MD simulation.

Complex	Average (kJ/mol)	Total drift (kJ/mol)
LJ-SR:Mpro(WT)- Boceprevir	-158.90	18.54
LJ-SR:Mpro(R60C)- Boceprevir	-134.43	-19.01
Coul-SR: Mpro(WT)- Boceprevir	-65.37	17.45
Coul-SR: Mpro(R60C)- Boceprevir	-59.56	6.49

## Discussion

Till date (April 23, 2020), 2.6 million cases of COVID-19 have been reported worldwide. In this study, we analyzed 13 complete sequences of SARS-CoV-2 reported from 13 different countries and compared with SARS-CoV. Phylogenetic analysis showed that SARS-CoV-2 sequences clustered together in a single clad irrespective of their geographic origin, whether from the same continent or neighboring countries. Replicase polyprotein, which are most conserved among coronaviruses, shared 87% amino acid sequence similarity to SARS-CoV, less than the threshold value (90%) for demarcation of betacoronavirus species [27]. They belong to new virus species *Severe acute respiratory syndrome-related coronavirus* of genus *Betacoronavirus* [28].

Among all known RNA viruses, coronaviruses consist of the largest genome (26.4 to 31.7 kb) [36, 37]. The large genome size provides more plasticity in accommodating and modifying genes [36–38]. Mutation frequency is very high in RNA viruses, which enhances virulence and responsible for the formation of new species [19]. The high frequency of mutation within the viral genome at different geographical locations may be one of the reasons that SARS-CoV-2 is responsible for change in mortality rate and symptom of the disease [39]. The comparison of amino acid sequences of replicase polyprotein of 13 SARS-CoV-2 showed mutations in India, China, South Korea, Sweden, Vietnam and Brazil strains at different amino acid locations. Earlier report showed the similar result i.e. a single mutation in replicase polyprotein at 3606 (L to F) [40]. We could identify few more single amino acid mutations at different positions in above mentioned SARS-CoV-2 strains (Table 1). The replicase polyprotein codes for nsp2 and nsp3 and it has been suggested in previous research that the mutation in nsp2 and nsp3 play a key role in infectious capability and are responsible for the differentiation mechanism of SARS-CoV-2 [39].

The RBD of spike protein is the region which specifically interact with ACE2 leading to viral entry into the host cell [41–43]. The Indian isolate of SARS-CoV-2 showed mutation within this region where at 408 position, Arginine is replaced by Isoleucine. For several years, the prediction of protein stability via theoretical or experimental approaches has been a profound area of research [44]. Earlier findings suggest that a single point mutation at RBD is responsible for disrupting the antigenic structure, thereby, affecting the binding of RBD to ACE2 [45, 46]. The mutation within this region of spike protein may affect the binding of RBD to its receptor, thus, affecting the viral entry within the host cells. Further, *in silico* studies revealed that this point mutation within the RBD of spike glycoprotein was having stabilization effect on spike protein and found to increase the protein stability (ΔΔG 0.49732107 kcal/mol).

Single amino acid mutation was observed in both Mpro (R60C) of SARS-CoV-2 Vietnam isolate and RdRp (A408V) of SARS-CoV-2 India isolate. The *in silico* findings revealed that the mutations in both strains decrease the stability of protein. The MD simulation studies on Mpro further confirmed that the point mutation on Mpro affects the stability of proteins as well as the binding of inhibitor. Our *in silico* study found that the catalytic active site of Mpro is surrounded by amino acid residues of a loop (142–145, 175–200), short helix (40–43, 46–50), and beta sheet regions (25–27, 164–167). The R60C mutant lies at helix adjacent to the short helix (H2) that forms the catalytic channel (Fig 5). Substitution of an amino acid with charged side chain to uncharged cysteine residue leads to loss of conserved ionic bond interaction and the effect cascades to other conserved ionic interactions. Loss of conserved ionic interaction was observed between amide nitrogen of arginine and carboxylic oxygen atom of aspartic acid at position 48 of the catalytic channel.

It was found that the short helix H2, that form the catalytic channel, have attained a more flexible loop like conformation in the mutant protein. Conserved hydrogen bonded interactions that stabilizes the catalytic channel L1 loop between Tyr54 OH⋯Asp187 Oδ1, Tyr54 OH⋯Asp187 O and Leu50 O⋯Arg188 NE were lost in the mutant enzyme, thereby, increasing the flexibility of structure forming the binding pocket (Fig 5B). Therefore, the local change of an ordered secondary structure to a more disordered loop like structure have increased the overall flexibility of the secondary structure elements forming the catalytic pocket, thereby, effecting the binding of ligand to residue in the catalytic channel. This is quite evident from the reduced LJ-SD and coulombic-SR interaction energies between the enzyme and ligand in wild and mutant complexes (Fig 4E and Table 7). The role of important active site residues, discussed in this study, has been reported before [47, 48]. The RMSF plot also reveals the key role of these residues in accommodating the inhibitor within the active site of Mpro.

Envelop protein plays an important role in the assembly of viral genome and the formation of ion channels (IC), responsible for virus-host interaction, which is mainly associated with pathogenesis [5, 49]. We detected one amino acid mutation L37H in transmembrane domain (TMD) of envelop protein of SARS-CoV-2 South Korea isolate. The TMD is hydrophobic in nature consisting mainly hydrophobic amino acids, while the mutation in TMD at 37 position changes hydrophobic to hydrophilic amino acid which changes the integrity of TMD. Earlier report showed that mutations within the TMD domain of envelop protein completely disrupted IC activity [50]. This might be one of the reasons for slow spreading/low pathogenicity of SARS-CoV-2 in South Korea.

Nucleocapsid protein of coronavirus is necessary for RNA replication, transcription and genome packaging [51, 52]. A mutation P344S in nucleocapsid protein has been detected in SARS-CoV-2 Japan strain. The P344S mutation on nucleocapsid was found to decrease the protein stability (ΔΔG -1.2252261). This mutation is located in carboxy-terminal RNA-binding domain (CTD) of nucleocapsid protein. Earlier studies showed that CTD is responsible for oligomerization [53].

It was also revealed that among all the genomes studied in this study, the Indian SARS-CoV-2 isolates were carrying maximum mutation. The Indian isolates were carrying the R408I on the spike protein while A406V on the RdRp and several mutations on the replicase polyprotein of SARS-CoV-2. It is expected that these large number of mutations among the SARS-CoV-2 may affect the vaccine/inhibitor development against these isolates.

## Conclusion

To conclude, SARS-CoV-2 complete sequences from 13 countries were analyzed and compared with SARS-CoV. We identified country specific mutations in major proteins (replicase polyprotein, spike protein, envelop protein and nucleocapsid protein). Further, molecular dynamics and other *in silico* studies revealed that mutations decrease the stability of protein and also hinders the binding of inhibitor. Mutation R408I in spike protein of Indian strain has significant influence on RBD domain of spike protein and this point mutation has a stabilization effect on the spike protein. The findings of the present study could help for the design of potential vaccine candidates/small molecular inhibitor against COVID19.

## Supporting information

S1 File (ZIP)

## Abbreviations

COVID-19: Coronavirus disease 19
MD: Molecular Dynamics simulations
MERS-CoV: Middle-East respiratory syndrome coronavirus
Mpro: Main protease
ORFs: Open reading frames
RdRp: RNA-dependent RNA polymerase
SARS-CoV: Severe acute respiratory syndrome coronavirus
SARS-CoV-2: Severe acute respiratory syndrome-related coronavirus-2
WT: Wildtype
